# A Functional Imaging Study of Self-Regulatory Capacities in Persons Who Stutter

**DOI:** 10.1371/journal.pone.0089891

**Published:** 2014-02-27

**Authors:** Jie Liu, Zhishun Wang, Yuankai Huo, Stephanie M. Davidson, Kristin Klahr, Carl L. Herder, Chamonix O. Sikora, Bradley S. Peterson

**Affiliations:** 1 Department of Psychiatry, The New York State Psychiatric Institute, Columbia College of Physicians and Surgeons, New York, New York, United States of America; 2 American Institute for Stuttering, New York, New York, United States of America; University of Texas Health Science Center at San Antonio, Research Imaging Institute, United States of America

## Abstract

Developmental stuttering is a disorder of speech fluency with an unknown pathogenesis. The similarity of its phenotype and natural history with other childhood neuropsychiatric disorders of frontostriatal pathology suggests that stuttering may have a closely related pathogenesis. We investigated in this study the potential involvement of frontostriatal circuits in developmental stuttering. We collected functional magnetic resonance imaging data from 46 persons with stuttering and 52 fluent controls during performance of the Simon Spatial Incompatibility Task. We examined differences between the two groups of blood-oxygen-level-dependent activation associated with two neural processes, the resolution of cognitive conflict and the context-dependent adaptation to changes in conflict. Stuttering speakers and controls did not differ on behavioral performance on the task. In the presence of conflict-laden stimuli, however, stuttering speakers activated more strongly the cingulate cortex, left anterior prefrontal cortex, right medial frontal cortex, left supplementary motor area, right caudate nucleus, and left parietal cortex. The magnitude of activation in the anterior cingulate cortex correlated inversely in stuttering speakers with symptom severity. Stuttering speakers also showed blunted activation during context-dependent adaptation in the left dorsolateral prefrontal cortex, a brain region that mediates cross-temporal contingencies. Frontostriatal hyper-responsivity to conflict resembles prior findings in other disorders of frontostriatal pathology, and therefore likely represents a general mechanism supporting functional compensation for an underlying inefficiency of neural processing in these circuits. The reduced activation of dorsolateral prefrontal cortex likely represents the inadequate readiness of stuttering speakers to execute a sequence of motor responses.

## Introduction

Developmental stuttering is a disorder of speech fluency that disrupts the rhythm and timing of speech, producing frequent repetitions, and prolongations and blocks of syllables and words. Stuttering symptoms follow a distinctive developmental trajectory, with speech dysfluency typically beginning in early childhood, then gradually attenuating with the development of language skills, and remitting by adolescence in approximately 75% of persons who stutter [1]. This distinctive course yields prevalence rates that vary with age, with approximately 5% of children and 1% of adults meeting diagnostic criteria for stuttering [1], [2]. The precise etiology of stuttering remains largely unknown, although recent neuroimaging studies have provided evidence for abnormal changes of volumes and function in widely distributed regions involved in the production of speech [3]–[14].

Prior fMRI studies of stuttering have focused nearly exclusively on assessing brain activation during tasks that involve speech processing or production in attempts to identify the neural circuits that produce stuttering. Studies using language-related tasks in persons who stutter, however, have been unable to distinguish the abnormalities in brain activation that contribute to generating speech dysfluencies in stuttering speakers from the activations that derive from the attempts to control those dysfluencies. Recent stuttering research has revealed impairments in capacities that can broadly be described as self-regulation, including regulation of attention, emotion, and motor activity [15]–[18]. Although these capacities may contribute to the maintenance and exacerbation of speech dysfluencies [16], [18], [19], prior neuroimaging studies have not yet assessed the functional integrity of self-regulatory systems. These systems likely modulate stuttering behaviors and thereby likely also determine the severity of symptoms and their persistence into adulthood. In addition, findings from studies in children have suggested that a compromised capacity of self-regulation may predispose to the development of stuttering [15], [19].

We therefore aimed to study self-regulatory control systems in the brains in stuttering speakers across the lifespan, from early childhood through adulthood, independent of the speech-based abnormalities that define stuttering behaviors. Self-regulatory functions are commonly studied using tasks in which participants must respond to a task-relevant feature of a stimulus for which processing is less automatic rather than a task-irrelevant feature of that same stimulus for which processing is more automatic. These differing features of the same stimulus produce cognitive interference [20]. We selected for use in this study the Simon Spatial Incompatibility task, one of a large class of self-regulatory tasks, because of the non-linguistic nature of its stimuli. A conflict in the Simon task is created when the less automatic, task-relevant response to the direction of an arrow stimulus conflicts with the more automatic, task-irrelevant (prepotent) response to the spatial location of the side of the screen on which the arrow is presented (e.g., when a leftward pointing arrow is presented on the right side of the screen). Correct responses in the conflict conditions require activation of frontostriatal circuits in order to mobilize controlled, less automatic responses elicited by the direction of the arrow and to inhibit more automatic responses elicited by the location of the arrow.

We hypothesized that we would detect evidence in clinically identified stutterers for functional compromise of self-regulatory control systems based within frontostriatal circuits of the brain. Similar to activation in various other childhood-onset behavioral disorders [21], we expected that stuttering speakers would activate frontostriatal circuits more strongly than would fluent control speakers in order to maintain performance during a task that requires behavioral control, and that stuttering speakers would activate frontal cortices more strongly in direct proportion to the severity of stuttering symptoms. In addition, some investigators have suggested that stuttering may be a consequence of a failed temporal integration of disparate speech segments into conversational speech sequences, thereby creating the interruptions and pauses that are the defining characteristic of dysfluent speech [22], [23]. We therefore also assessed the influence of temporal context on behavioral performance and brain activation in stuttering speakers during this self-regulatory task.

## Materials and Methods

### Participants

We recruited 46 participants with developmental stuttering through advertisements posted online and at local clinics, hospitals, and stuttering support groups. All participants in the stuttering group had been diagnosed by a licensed speech-language pathologist before enrollment in the study. Fifty-two fluent controls to match for age and sex were recruited randomly from a telemarketing list of 10,000 names in the local community, excluding those with lifetime Axis I disorders, or any language disorders.

We administered the Kiddie-Schedule for Affective Disorders and Schizophrenia, Present and Lifetime Version for participants under age 18 [24], and the Structured Clinical Interview for DSM-IV-TR Axis I Disorders for those over 18 [25], to establish diagnoses of comorbid disorders in stuttering speakers. Five stuttering participants had one comorbid disorder, including chronic motor tic disorder (N = 2), attention deficit hyperactivity disorder (N = 2), and social anxiety disorder (N = 1). We allowed into the study stuttering participants who had comorbidities because these co-occurring illnesses are highly prevalent in stuttering speakers; excluding comorbidities would have impaired the generalizability of our findings to the larger population of stuttering speakers from which our sample was drawn. Stuttering severity was evaluated on the day of the scan by the Assessment of the Child’s Experience of Stuttering (ACES) for children who stuttered and the Overall Assessment of the Speaker’s Experience of Stuttering (OASES) for adults who stuttered [26], [27]. Severity ranged from mild to moderate-to-severe, with the majority of stuttering participants (70%) endorsing at least moderate level of symptoms. Moreover, IQ was evaluated by the Wechsler Abbreviated Scale of Intelligence [28].

Additional exclusionary criteria for all participants included a history of premature birth, prior head trauma with loss of consciousness, past seizures, mental retardation, pervasive developmental disorders, or chronic medical illness. Study procedures were approved by the Institutional Review Board of the New York State Psychiatric Institute. Written informed consent was obtained from all adult participants and the parents of child participants. Children also provided written or verbal (at or younger than 6 years) assent for their participation.

### fMRI Paradigm

**Figure 1:**
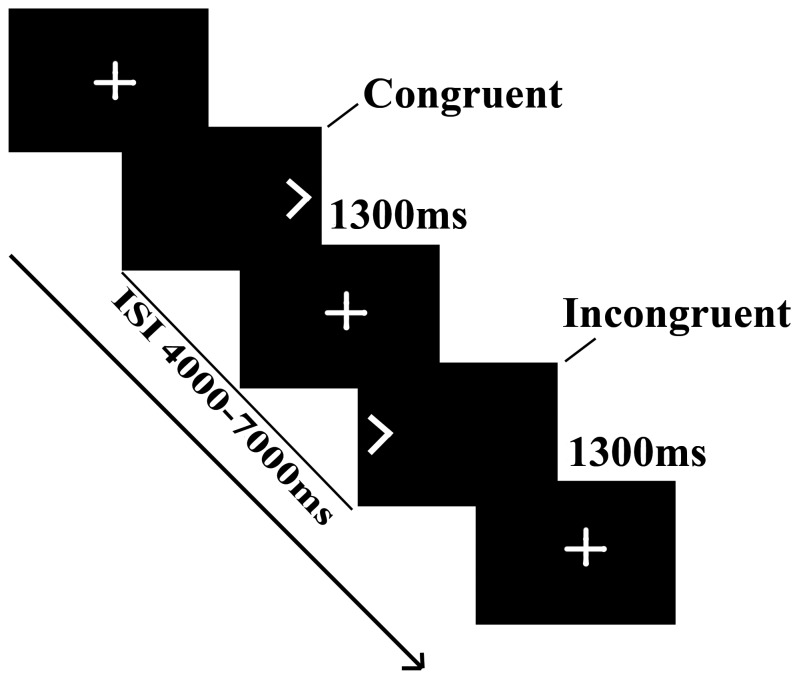
Experimental design of the Simon Spatial Incompatibility Task.

We used the Simon Spatial Incompatibility Task to investigate alterations of self-regulatory functions in stuttering [29]. Participants in each trial viewed a leftward or rightward pointing arrow that was either consistent or inconsistent with the side of the screen (left or right) on which the arrow was displayed. Participants were instructed to respond as quickly as possible without making errors to the direction in which the arrow was pointing by pressing a button on a response box, with the right index finger for a left-pointing arrow and the right middle finger for a right-pointing arrow. The arrow stimulus was congruent (pointing in the same direction as its position relative to the midline of the screen) or incongruent (pointing in the opposite direction to its position), based on congruency between the direction and position feature of the stimulus (Fig. 1). This event-related paradigm comprised three runs. Forty-four stimuli (22 congruent and 22 incongruent, with direction/position counterbalanced) were displayed in each run in a pseudorandom order with a fixed duration of 1300 msec. Stimuli were separated by interstimulus intervals (ISI) of jittered durations (range 4000–7000 msec; mean 5372±840.7 msec) to prevent expectancy effects. A fixation cross-hair was presented in the center of the screen during ISIs. The task was programmed and run by E-Prime software (http://www.pstnet.com). The reaction time (RT) and accuracy of each button-press were recorded.

### Image Acquisition

Images were collected on a 3-Telsa GE Signa EXCITE scanner (General Electric, Milwaukee, USA). Head positioning was standardized using the canthomeatal line. A sagittal T1-weighted localizing scan was obtained to determine anterior-posterior commissure line, to which the axial functional images were aligned. Following the localizing scan, the functional images were acquired using a T2*-weighted single-shot echo-planar imaging sequence with repetition time 2200 msec, echo time 30 msec, image matrix 64×64, field of view 24×24 cm, flip angle = 90°, 34 slices, slice thickness 3.5 mm without gap, 140 volumes collected per run. Each run lasted 5 minutes 8 seconds, totaling 15 minutes 24 seconds for three runs.

### Image Processing

Image processing was performed using Statistical Parametric Mapping software (http://www.fil.ion.ucl.ac.uk/spm). Preprocessing procedures consisted of spatial realignment, slice timing correction, normalization to the standard template in Montreal Neurological Institute space, and smoothing with a 8 mm full-width-at-half-maximum Gaussian kernel. A high pass filter (1/128 Hz cutoff) was applied subsequently to remove low-frequency noise from the fMRI time series.

#### Subject-level analyses

A subject-level analysis was performed in each participant in the context of a general linear model for statistical estimation. Analyses focused only on correct trials denoted by combinations of congruency of preceding trial (congruent or incongruent) and current trial. Each event of interest was modeled by its time series convolved with the canonical hemodynamic response function. Incorrect trials, omitted trials, and RT outliers were modeled as regressors of no interest and excluded from further analyses. Linear contrasts of event regressors were constructed to estimate neural activity corresponding to conflict resolution and context-dependent adaptation.

#### Conflict resolution

Resolving conflict in this task requires engagement of frontostriatal circuits to enact a controlled response to direction while withholding the automatic response tendency to position of the arrow stimulus. We compared brain activation between “cI” trials (i.e., incongruent trials immediately preceded by a congruent trial, which are high in conflict and typically produce the longest RTs) and “cC” trials (i.e., congruent trials immediately preceded by a congruent trial, which are lowest in conflict and produce the shortest RTs) so as to identify neural systems involved in resolving conflict.

#### Context-dependent adaptation

A well-replicated observation in this task is that RTs shorten during trains of repeated stimuli, so that RTs to “iI” trials (i.e., incongruent trials immediately preceded by an incongruent trial) are shorter than RTs to “cI” trials (i.e., incongruent trials immediately preceded by a congruent trial), even though the current stimulus in both trials is incongruent. Improved responses to current conflict following a previous encounter of a conflict is thought to stem from the self-regulatory function integrating the stimulus context from the recent past with the current stimulus (i.e., adaptive responses to the current incongruent are contingent on the conflict content of immediately preceding stimulus). We used the iI vs cI contrast to assess the functional integrity of brain regions in mediating these cross-temporal contingencies, a crucial function in the execution of motor and linguistic sequences.

#### Group-level analyses

Contrast maps were further entered into a group-level analysis, in which diagnosis (stutterers or controls) was considered as a random effect. Two-sample t-tests were applied to assess group differences in conflict resolution and context-dependent adaptation while covarying for age, sex, IQ scores, and RT interference (the difference in mean RTs for incongruent and congruent stimuli). Furthermore, we correlated parameter estimates of task-related activation with total score (standardized by z-transform) of ACES/OASES subscaleIII (Communication in Daily Situations), the most representative index of speech difficulties in various social settings. Results from voxel-based analyses were thresholded at a p-value<0.025 for individual voxels and a spatial extent of at least 30 voxels (p<0.05, cluster-level corrected based on Monte Carlo simulations) [30].

#### Functional connectivity analyses

We also performed an analysis of effective connectivity using Granger causality. The Granger Causality Index of A on B, denoted by (GCI_(A−>B)_), measures the predictability of current values of B from previous values of A. In fMRI time series analyses, the mathematical definition of GCI = 1-var(eAB)/var(eB) produces positive results when the variance of error in prediction of current B values using previous values of A and B (var(eAB)) is reduced compared to that using B previous values alone (var(eB)) in an autoregressive model [31]–[33]. Our connectivity analyses focused on how the activation in anterior cingulate cortex and dorsolateral prefrontal cortex causally influenced one another during performance of the Simon task, and whether strength of these directed influences was aberrant in stuttering speakers.

## Results

### Sample Characteristics

Stuttering speakers and fluent controls did not differ significantly in age or sex distribution, though controls had slightly higher IQ scores than did stuttering participants (Table 1).

**Table 1:** Participants Characteristics.

	Participants		
Characteristic	Stutterers (N = 46)	Controls (N = 52)	*P* Value
Age Range, yrs	5 to 51	6 to 50	
Age, yrs (Mean±SD)	24.0±11.0	23.1±11.5	0.70
WASI IQ (Mean±SD)^a^	108.2±2.11	115.5±2.10	0.02
Sex (M%)	63%	64%	0.97
Ethnicity [n(%) Caucasian]	18(39%)	34(65%)	
Stuttering Severity (n child: n adult)^b^			
Mild	1∶0		
Mild-to-Moderate	7∶5		
Moderate	9∶13		
Moderate-to-Severe	2∶6		
Severe	0∶0		
Taking Medications, n(%)^c^	10(22%)		

Abbreviations: WASI, Wechsler Abbreviated Scale of Intelligence.

a WASI IQ score was missing in one healthy control.

b Severity was based on the Impact Rating from the Assessment of the Child’s Experience of Stuttering for child stutterers and the Overall Assessment of the Speaker’s Experience of Stuttering for adult stutterers. Severity ratings were missing in one child and in two adult stutterers.

c 7 stuttering speakers on antidepressants, 2 on benzodiazepines, and 3 on stimulant medications at the time of the scan. Medications were not mutually exclusive.

### Behavioral Performance

**Figure 2:**
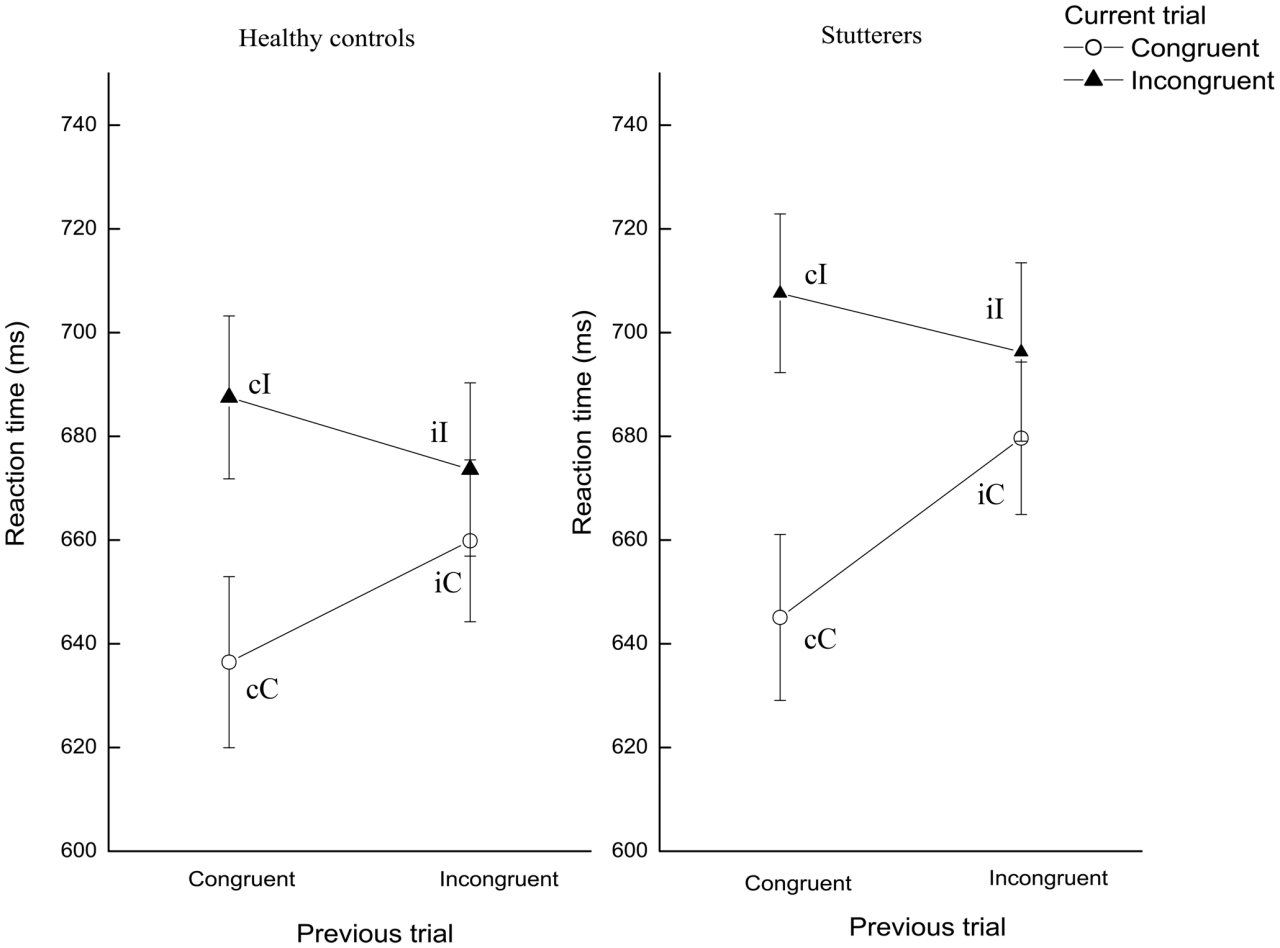
Behavioral results. Mean reaction times (± standard errors) of stutterers and healthy controls for current trial congruency were plotted under modulation of preceding trial congruency. Abbreviations: cC = congruent trials immediately preceded by a congruent trial; cI = incongruent trials immediately preceded by a congruent trial; iC = congruent trials immediately preceded by an incongruent trial; iI = incongruent trials immediately preceded by an incongruent trial.

Behavioral performance was analyzed using a repeated measures analysis of variance with factors of congruency(incongruent, congruent) of preceding and current trial and diagnosis (Fig. 2). We detected a significant main effect of current trial congruency (F_1,96_ = 65.4, p<0.01, partial η^2^ = 0.41), but no interactions with diagnosis (diagnosis×current trial congruency, F_1,96_ = 0.65, p = 0.42), indicating that stuttering speakers and controls both required more time to respond to incongruent than to congruent stimuli. Furthermore, response latency on the current trial was modulated by congruency of the previous trial, as represented by a significant interaction of current trial and preceding trial congruency (F_1,96_ = 40.51, p<0.01, partial η^2^ = 0.30). Post hoc analyses indicated that this interaction derived from responses to iI stimuli that were faster than to cI stimuli (t_97_ = 3.16, p = 0.002), or from context-dependent adaptation. The absence of significant three-way interactions (preceding trial congruency×current trial congruency×diagnosis, F_1,96_ = 0.43, p = 0.51) indicated that both groups similarly speeded their RTs to incongruent-incongruent stimulus trials. Both groups maintained a high level of accuracy throughout the task (>70% across three runs); groups did not differ in accuracy during incongruent (t_96_ = −0.56, p = 0.57) or congruent trials (t_96_ = −0.91, p = 0.36).

### Brain Activation and Connectivity

**Figure 3:**
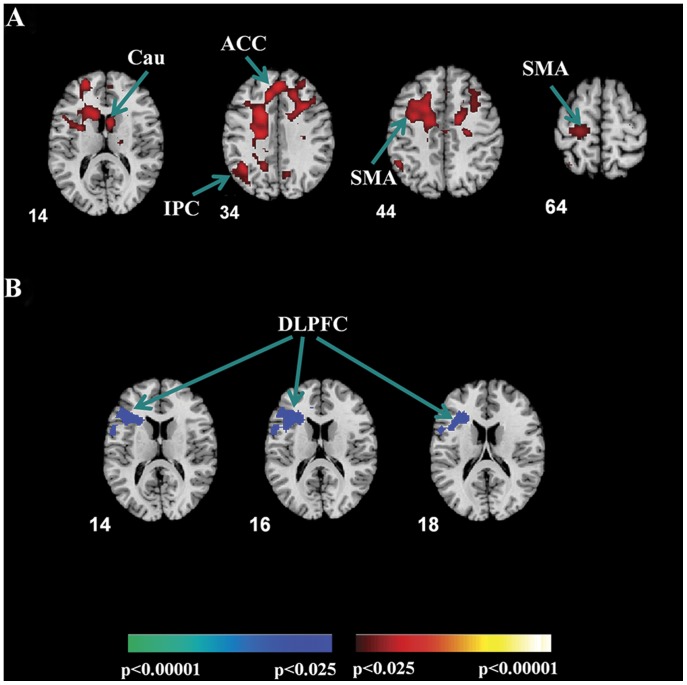
Activation Maps. (A) Representative axial–oblique slices depict greater activation of stutterers relative to healthy controls in frontostriatal regions when resolving Simon conflict (activation contrast: incongruent trials immediately preceded by a congruent trial versus congruent trials immediately preceded by a congruent trial). Abbreviations: ACC = anterior cingulate cortex; Cau = caudate; IPC = inferior parietal cortex; SMA = supplementary motor area. (B) Axial–oblique views of attenuated activation in the dorsolateral prefrontal cortex of stutterers as compared to healthy controls during response to incongruent trials under the influence of congruency of preceding trial (context-dependent adaptation, activation contrast: incongruent trials immediately preceded by an incongruent trial versus incongruent trials immediately preceded by a congruent trial). Abbreviations: DLPFC = dorsolateral prefrontal cortex. Statistical maps were displayed at a threshold of p<0.025 with a cluster extent of 30 voxels (P<0.05, corrected). The left-hand sides of the images correspond to the left side of the brain. The color bars indicate the t-values. The coordinates in Montreal Neurological Institute space are defined in millimeters.

Neural activity indexing conflict resolution was examined using the cI-cC contrast. Greater activation was detected in stuttering speakers in the anterior cingulate cortex (ACC), left anterior prefrontal cortex, right medial frontal cortex (MFC), left supplementary motor area (SMA), right caudate nucleus, and left parietal cortex (Table 2, Fig. 3A).

**Table 2:** Brain Regions of Group-Level Differences during Performance of the Simon Task.

Brain region	Location			MNI coordinates			T statistic
	Side	BA	No. of voxels	x	y	z	
*Stutterers>fluent controls during conflict resolution*							
Inferior parietal cortex	L	BA39	150	−41	−58	34	3.03
Anterior prefrontal cortex	L	BA10	131	−23	53	18	2.60
Medial frontal cortex	R	BA9	206	1	39	31	2.58
Supplementary motor area	L	BA6	135	−22	−22	64	2.43
Posterior cingulate cortex	R	BA23	61	1	−56	20	2.38
Anterior cingulate cortex	L	BA32	184	−6	28	31	2.09
Caudate	R		57	11	6	14	2.07
*Stutterers<fluent controls during context-dependent adaptation*							
Dorsolateral prefrontal cortex	L	BA46	88	−48	30	16	−2.68
	L	BA44	74	−54	12	15	−2.40
	L	BA8	126	−46	10	40	−2.33
	L	BA45	70	−53	23	14	−2.30

Abbreviations: BA, Brodmann area; L, left; MNI, Montreal Neurological Institute; R, right.

Neural activity associated with context-dependent adaptation was assessed by comparing BOLD signal between iI and cI trials, revealing decreased activation of the dorsolateral prefrontal cortex (DLPFC) in stuttering speakers compared with controls (Table 2, Fig. 3B). No brain regions were detected in which stuttering group showed increased activation.

**Figure 4:**
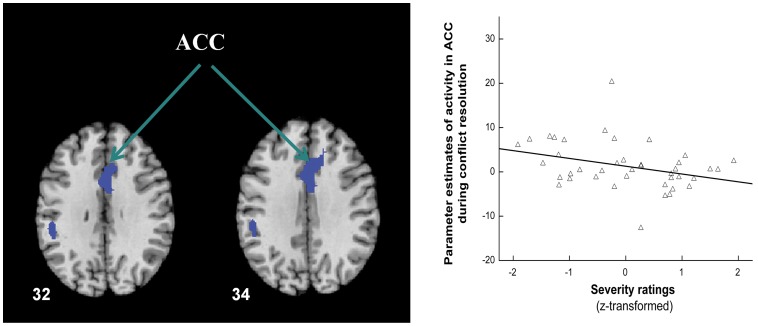
Severity correlation. Axial–oblique views of brain regions in stutterers show negative correlations between the magnitude of brain activation during conflict resolution and stuttering severity as measured with the ACES and OASES. The scatter plot illustrates the negative association in the ACC between the standardized severity score and the parameter estimate of brain responses during conflict resolution (r = −0.33, p<0.025). Abbreviations: ACC = anterior cingulate cortex.

The magnitude of activation in stuttering speakers during conflict resolution correlated inversely with score of ACES/OASES subscaleIII in the anterior cingulate cortex (Fig. 4), left insular cortex, and bilateral inferior parietal lobule (Table 3).

**Table 3:** Regions of Significant Correlation between fMRI Activation during Conflict Resolution and Severity of Stuttering^a^.

Brain region	Location			MNI coordinates			T statistic
	Side	BA	No. of voxels	x	y	z	
Inferior parietal lobule	R	BA40	130	63	−43	22	−3.33
Insular cortex	L	BA13	83	−39	14	7	−3.16
Inferior parietal lobule	L	BA40	107	−51	−31	28	−2.88
Anterior cingulate cortex		BA32	77	0	18	34	−2.62

Abbreviations: BA, Brodmann area; L, left; MNI, Montreal Neurological Institute; R, right.

a Severity of stuttering was based on the z-transformed score on the subscaleIII (Communication in Daily Situations) of the Assessment of the Child’s Experience of Stuttering (ACES) for child stutterers and the Overall Assessment of the Speaker’s Experience of Stuttering (OASES) for adult stutterers.

Granger causality analyses revealed in both stuttering and fluent speakers significant causal influences of the DLPFC on the ACC (GCI_(DLPFC->ACC)_) and vice versa (GCI_(ACC->DLPFC)_). Bidirectional GCIs remained significant after Bonferroni adjustment for multiple comparisons. The strength of causal influences, in contrast, did not differ between the two groups in any causality metric (Table 4).

**Table 4:** Granger Causality Analyses for the Anterior Cingulate Cortex and Dorsolateral Prefrontal Cortex during Performance of the Simon Task.

	Stutterers (N = 46)†	Controls (N = 52)†	Stutterers vs.Controls††
**ACC->DLPFC(BA46)**	0.034±0.04, p = 2.43E-6	0.029±0.03, p = 4.13E-8	z = 0.110, p = 0.91
**ACC->DLPFC(BA44)**	0.032±0.04, p = 2.15E-7	0.025±0.04, p = 6.50E-9	z = 1.107, p = 0.27
**ACC->DLPFC(BA8)**	0.023±0.03, p = 6.59E-6	0.033±0.05, p = 5.41E-9	z = 0.609, p = 0.54
**ACC->DLPFC(BA45)**	0.035±0.04, p = 1.39E-7	0.021±0.04, p = 1.52E-8	z = 0.698, p = 0.49
**DLPFC(BA46)->ACC**	0.016±0.02, p = 8.94E-6	0.021±0.04, p = 1.96E-4	z = −0.046, p = 0.96
**DLPFC(BA44)->ACC**	0.024±0.02, p = 2.83E-7	0.025±0.04, p = 5.76E-5	z = 0.680, p = 0.50
**DLPFC(BA8)->ACC**	0.022±0.03, p = 3.95E-6	0.033±0.05, p = 3.00E-6	z = −0.666, p = 0.51
**DLPFC(BA45)->ACC**	0.019±0.02, p = 1.31E-6	0.021±0.04, p = 2.21E-4	z = 1.0325, p = 0.31

Abbreviations: ACC, anterior cingulate cortex; BA, Brodmann area; DLPFC, dorsolateral prefrontal cortex.

† Granger causality indices (mean±standard deviation) of functional connectivity between the ACC and DLPFC subregions, including BA46, 44, 8, and 45. The ACC seed region was an 8 mm-radius sphere centered at the voxel (x = −6, y = 28, z = 31) in which stuttering speakers activated more strongly relative to fluent speakers during conflict resolution. The DLPFC seed regions were defined using 8 mm spheres placed at the foci of activation (BA46, x = −48, y = 30, z = 16; BA44, x = −54, y = 12, z = 15; BA8, x = −46, y = 10, z = 40; BA45, x = −53, y = 23, z = 14) in which stuttering speakers showed blunted activation during context-dependent adaptation.

†† Between-group comparisons of Granger causality indices.

### Potential Confounds

To clarify the confounding contributions of use of medications among stuttering speakers, we performed a subgroup analysis excluding 10 stuttering speakers who were taking medications at the time of the scan. Comparisons between nonmedicated stuttering speakers and controls yielded similar results to those reported when including the entire study population, except that the severity correlations in ACC diminished in significance, presumably a consequence of lowered statistical power (Fig. S1). We also entered medication use as a covariate in analyses that included the entire stuttering group, yielding unchanged results as well. Similarly, statistical covariation for comorbid psychiatric illnesses and ethnicity (Fig. S2) did not alter group differences in brain activation. These findings suggest that medication use, comorbid illnesses, and ethnicity were not the causes of the group differences in brain activation that we observed.

## Discussion

Although stuttering and control participants performed similarly on a self-regulatory task, stuttering speakers activated frontostriatal regions more strongly when resolving conflict. These regions included the ACC, anterior PFC, SMA, MFC, parietal cortex, and caudate. The heightened activations presumably reflected a need for greater mobilization of self-regulatory functions in order to maintain normal performance in stuttering speakers while withholding automatic responses to conflict-laden stimuli. Activation of the ACC correlated inversely with the severity of stuttering symptoms. In contrast, activation while adapting to changes in conflict was blunted in the DLPFC of stuttering speakers compared with control participants.

The resolution of conflict typically activates in healthy persons a widely distributed set of brain regions, including the ACC, DLPFC, SMA, parietal and temporal cortices, and striatum [34]. Neurocomputational models suggest that the ACC functions as a central hub for conflict resolution by evaluating the presence of conflict [35] and then orchestrating the functions of multiple regions that participate in resolving conflict. The anterior PFC and parietal regions support the allocation of attentional resources to the spatial locations of targeted stimuli [36], [37]. The SMA organizes motor sequences and regulates motor responses [38]. The striatum implements both feed-back and feed-forward controls to ensure successful realization of motor planning [39].

Regions that activated more in stuttering speakers were the ones that fluent control participants recruited during conflict resolution [34]. The substantial overlap of activations across groups suggests that the stuttering speakers engaged the same neural network as controls, but to a greater extent. Functional alternations in frontostriatal circuits have been noted consistently as compensatory responses across numerous developmental disorders [21]. We thus speculate that greater activation of the frontostriatal system in stuttering speakers similarly compensates for the presence of neural inefficiency in frontal regions when resolving conflict.

The analysis of activations during temporal context-dependent adaptation complemented the analysis of activations during conflict resolution by specifically assessing neural activity that supports the changes in self-regulation across time, particularly the contingency between the presence of a conflict-laden stimulus in the immediate past with the improved response to conflict in the present moment. In contrast to the greater activation of frontostriatal circuits during conflict resolution in stuttering speakers, activation during temporal context-dependent adaptation was much stronger in the DLPFC in the control participants than in the stuttering participants. The DLPFC transforms sensory percepts of stimuli and their temporal contexts into abstract representations, keeping them on-line and integrating them into current decisions for motor response [40]. Single-cell recordings in non-human primates have shown that lesions to the DLPFC disrupt the capacity to adapt to conflict by damaging two subpopulations of DLPFC neurons, those encoding the current conflict and those encoding the immediately preceding conflict [41]. Involvement of DLPFC in mediating cross-temporal contingencies and the anticipation of upcoming conflict prepares for a faster response in the following conflict trial by enhancing the levels of motor readiness [40]. We therefore interpret the blunted DLPFC activation in stuttering speakers as representing poor efficiency of the DLPFC in mediating adaptive cross-temporal contingencies for self-regulatory functions in persons who stutter.

Fluent speech requires smooth transitions from one segment of speech to the next. Motor and lexical programming for the forthcoming segment is under way during utterance of the present segment. The sequential motor and lexical processing for adjacent speech segments therefore overlaps one another in time. Dysfluency in stuttering speakers has been posited to originate from a difficulty in concatenating speech segments in a temporally coordinated manner [22]. Our analyses of temporal context-dependent adaptation enabled us to study self-regulatory control over the segment-to-segment transitions in motor planning during the sequential processing of conflict-laden stimuli and then associate that control with the impairment associated with stuttering symptoms. Inefficient functioning of the DLPFC would reduce motor readiness for processing of subsequent stimuli and, presumably, sequential speech segments. DLPFC dysfunction may also be involved in the reported difficulty that stuttering speakers have with the learning of motor sequences [23].

The modular processes of conflict resolution and adaptation interact in a cooperative manner through the mutual interconnections of neural circuits that support each of these processes. The prevailing theory for these circuit-based functions is that the ACC first detects the presence of conflict and then signals its presence to the DLPFC, which in turn helps to regulate adaptation to subsequent conflict [42], [43]. These hypothesized roles for the ACC and DLPFC and their interactions suggest that blunted activation of the DLPFC may make persons who stutter less able to maintain adequate performance during the subsequent adaptation to conflict and in mediating the cross-temporal contingencies that regulate behavior. An expedient solution to address this propensity for weakened activity of the DLPFC is to augment activation of other components of conflict processing, particularly the regulatory functions of the ACC. Thus, blunted activation of the DLPFC during conflict adaptation in stuttering speakers could be the driving force for heightened ACC activation during conflict resolution.

Our finding that stuttering severity correlated inversely with the magnitude of ACC activation during conflict resolution further supports our interpretation that heightened ACC activation plays a compensatory role in the pathogenesis of stuttering. Greater ACC-dependent regulation of behavior presumably supports better speech fluency, presumably by compensating for the poor efficiency of the DLPFC in mediating cross-temporal behavioral contingencies in persons who stutter. Finally, Grange Causality Indices were similar in stuttering speakers to those in fluent speakers, indicating the presence of normal functional communications between the ACC and DLPFC in stuttering speakers. In the presence of normal functional communication with the ACC, blunted DLPFC activation during context-dependent adaptation (representing inadequate preparation in the prior trial for the forthcoming conflict stimulus in the current trial) would be expected to generate compensatory activation of the ACC to support correct behavioral responding to conflict in the current trial.

Brain regions that subserve self-regulatory processes in the Simon task, including the ACC, adjacent motor areas, and DLPFC, also support the production of normal speech [44]–[48], and we hypothesize that disturbances in self-regulatory control over speech functions contribute importantly to dysfluent speech in persons who stutter. Consistent with this hypothesis, prior studies have identified in stuttering speakers reduced blood flow to the ACC at rest and abnormalities of functional connectivity in motor cortices, basal ganglia, Broca’s area, and neighboring regions during speech tasks [4], [10], [12], [49]. Therefore, we suspect that the functional abnormalities we observed in the ACC and DLPFC during the Simon task likely represent disturbances in self-regulatory capacities that contribute to dysfluent speech in persons who stutter. Altered capacities for self-regulation could conceivably contribute to the other myriad manifestations of stuttering as well. They may help to explain, for example, reports of motor control deficits and exaggerated stress responses in stuttering persons [19], [23]. Blunted activation of the DLPFC in adapting to conflict on a trial-by-trial basis may help account for the otherwise puzzling reported absence of practice-induced improvements in reaction time in stutterers when learning motor sequences [23]. Finally, poor regulatory control over emotions is thought to contribute to the worsening of stuttered speech in stressful speech situations [19].

Our study has several limitations. First, the range of ages of the participants was wide, potentially increasing the variance of brain activations. We covaried for age in all our analyses, however, and we additionally detected no age-dependent effects on our findings, suggesting that the findings obtain for all ages in the sample, consistent with findings from prior behavioral studies that impaired capacities are present throughout development [15], [19]. Moreover, we included participants over a wide age range by design to permit a developmental assessment of brain activation in stuttering speakers: the absence of age-dependent effects suggests that group differences in brain activations likely may represent a trait-like biomarker for stuttering speakers of all ages. Second, the slightly lower IQ scores in the stuttering group potentially could contribute to differences in brain activation as a consequence of cognitive underperformance in stuttering participants. The small group differences in IQ seem unlikely to have influenced our findings, however, given that the stuttering participants had IQs that were above average (mean of 108), stuttering participants performed equally well as controls on the task, and IQ was included as a covariate in our analyses. Third, comorbid illnesses and the use of psychotropic medications in stuttering participants could have influenced our results. Findings from analyses that included comorbidity and medications as dichotomized covariates and that excluded from the analyses participants who were taking medication, however, were essentially unchanged from those in analyses of the entire sample that did not include these covariates. Fourth, the use of the ACES/OASES self-reports as measures of stuttering severity did not provide objective measures of stuttering severity. We selected these instruments because they assess perceived control over stuttering symptoms, which is a construct most closely related to the capacity for self-regulation, the construct we were explicitly studying. Future studies should include both self-reports and objective measures of speech fluency. Fifth, diverse ethnicity in the study population could have biased our findings, although controlling for ethnicity in our analyses produced the same results as when not controlling for it. Finally, our findings are derived from a non-language task, and therefore their implications for speech production need to be tested directly in language-based tasks, especially with the aid of acoustic analyses of segment-to-segment transitions to assess whether cross-temporal contingencies in self-regulatory control do indeed relate to difficulties with the segmentation of speech over time.

Conventional theories tend to conceptualize stuttering as a discrete symptom confined mostly to the domain of language. The limitations of this singular focus on studying disturbances in language systems are clear when considering the robust abnormalities in neural responses of persons who stutter in our non-language task, especially the strong correlations of activation with stuttering severity. Our findings suggest that the pathogenesis of stuttering involves more than speech dysfluency. In particular, altered capacities for self-regulation may be important in determining the severity of symptoms. In addition, our assessment of the neural support for context-dependent adaptation was intended to probe processes that may also support the dynamic regulation of speech fluency across time. Reduced DLPFC activation during cross-temporal adaptation may therefore also contribute to the difficulty that stuttering speakers have in binding speech segments into fluent sequences across time. Inefficient DLPFC functioning presumably produces a compensatory heightened activation of the ACC and related frontal regions to support better behavioral regulation in persons who stutter.

## Supporting Information

Figure S1 (A) Stronger activation in frontostriatal regions during conflict resolution of stuttering speakers who were not taking psychoactive medications relative to fluent speakers (activation contrast: incongruent trials immediately preceded by a congruent trial versus congruent trials immediately preceded by a congruent trial, corrected P<0.05, cluster size >30). (B) Blunted activation in the dorsolateral prefrontal cortex during context-dependent adaptation of stuttering speakers who were not taking psychoactive medications relative to fluent speakers (activation contrast: incongruent trials immediately preceded by an incongruent trial versus incongruent trials immediately preceded by a congruent trial, corrected P<0.05, cluster size >30).
(TIF)

Figure S2 (A) After controlling for ethnicity, activation remains stronger in frontostriatal regions of stuttering speakers during conflict resolution relative to fluent speakers (activation contrast: incongruent trials immediately preceded by a congruent trial versus congruent trials immediately preceded by a congruent trial, corrected P<0.05, cluster size >30). (B) Activation remains blunted in the dorsolateral prefrontal cortex of stuttering speakers during context-dependent adaptation after controlling for ethnicity (activation contrast: incongruent trials immediately preceded by an incongruent trial versus incongruent trials immediately preceded by a congruent trial, corrected P<0.05, cluster size >30).
(TIF)
