# Supplementary figures and images for: A Functional Imaging Study of Self-Regulatory Capacities in Persons Who Stutter
Source: PLoS One. 2014 Feb 27;9(2):e89891. doi: 10.1371/journal.pone.0089891 (PMC3937393; doi:10.1371/journal.pone.0089891)

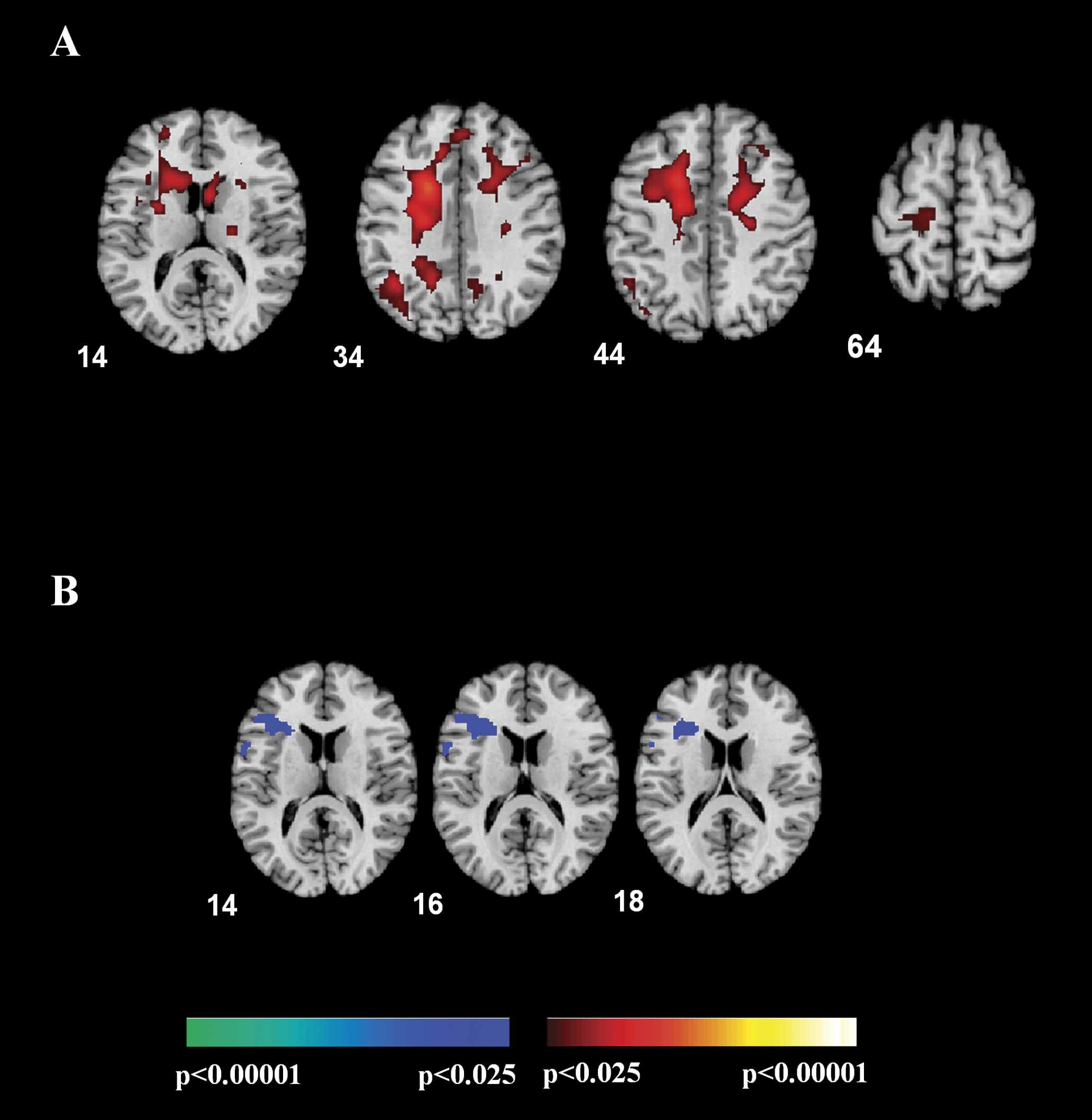

Supplement: Figure S1 — (A) Stronger activation in frontostriatal regions during conflict resolution of stuttering speakers who were not taking psychoactive medications relative to fluent speakers (activation contrast: incongruent trials immediately preceded by a congruent trial versus congruent trials immediately preceded by a congruent trial, corrected P<0.05, cluster size >30). (B) Blunted activation in the dorsolateral prefrontal cortex during context-dependent adaptation of stuttering speakers who were not taking psychoactive medications relative to fluent speakers (activation contrast: incongruent trials immediately preceded by an incongruent trial versus incongruent trials immediately preceded by a congruent trial, corrected P<0.05, cluster size >30). (TIF) [file pone.0089891.s001.tif]

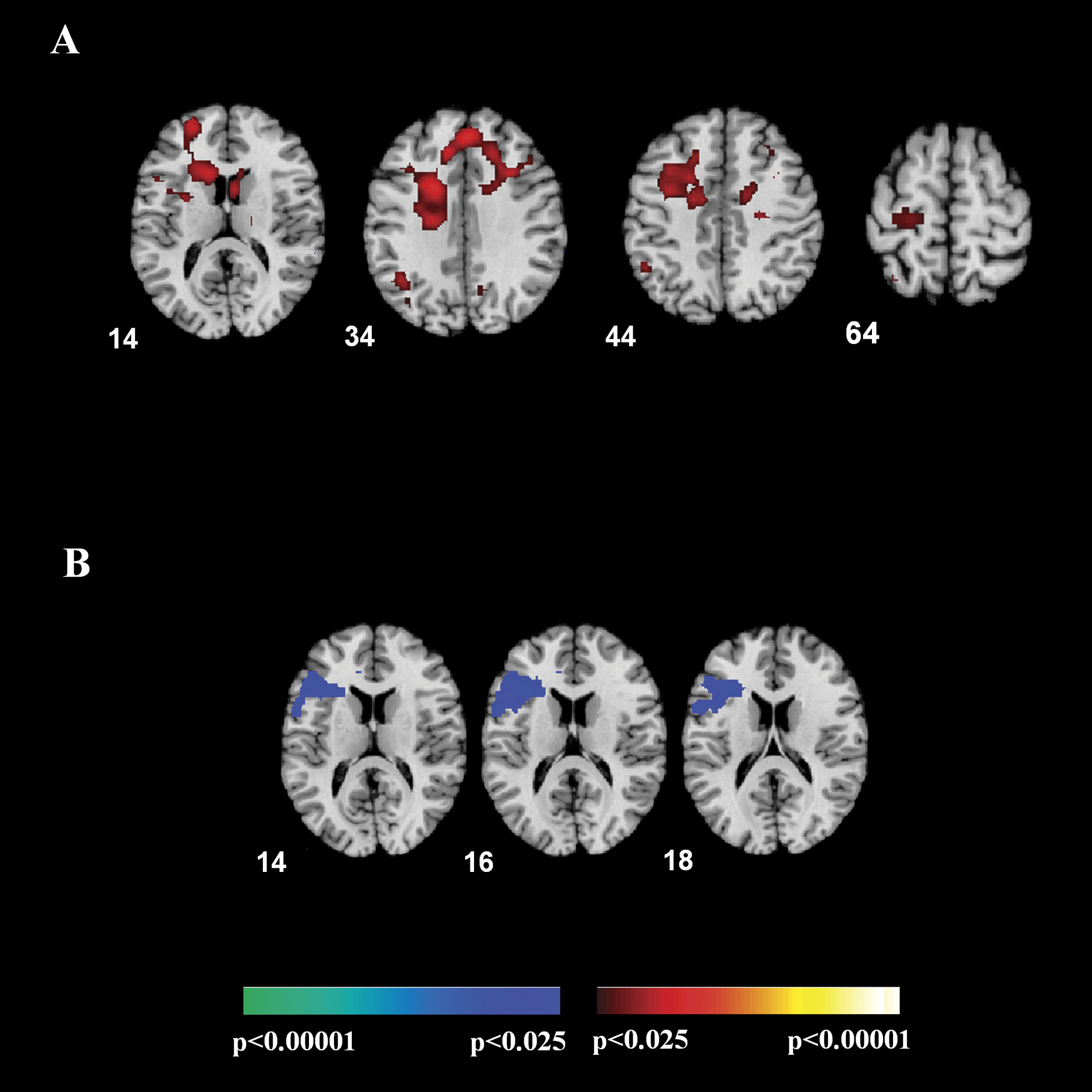

Supplement: Figure S2 — (A) After controlling for ethnicity, activation remains stronger in frontostriatal regions of stuttering speakers during conflict resolution relative to fluent speakers (activation contrast: incongruent trials immediately preceded by a congruent trial versus congruent trials immediately preceded by a congruent trial, corrected P<0.05, cluster size >30). (B) Activation remains blunted in the dorsolateral prefrontal cortex of stuttering speakers during context-dependent adaptation after controlling for ethnicity (activation contrast: incongruent trials immediately preceded by an incongruent trial versus incongruent trials immediately preceded by a congruent trial, corrected P<0.05, cluster size >30). (TIF) [file pone.0089891.s002.tif]
